# Effects of air pollution and seasonality on the respiratory symptoms and health-related quality of life (HR-QoL) of outpatients with chronic respiratory disease in Ulaanbaatar: pilot study for the comparison of the cold and warm seasons

**DOI:** 10.1186/s40064-016-3481-x

**Published:** 2016-10-19

**Authors:** Motoyuki Nakao, Keiko Yamauchi, Yoko Ishihara, Bandi Solongo, Dashtseren Ichinnorov

**Affiliations:** 1 Department of Public Health, School of Medicine, Kurume University, 67 Asahimachi, Kurume, Fukuoka 830-0011 Japan; 2 Department of Respiratory Medicine, Mongolian National University of Medical Sciences, Ulaanbaatar, Mongolia

**Keywords:** Chronic obstructive pulmonary disease, Bronchial asthma, Air pollution, Mongolia, PM2.5, HR-QoL, SF-36, COOP/WONCA charts

## Abstract

**Background:**

This study was performed to investigate the effects of air pollution and seasonality on the respiratory symptoms and health-related quality of life (HR-QoL) of outpatients with respiratory diseases in Ulaanbaatar, Mongolia. Subjects were outpatients who visited the hospital with chronic obstructive pulmonary diseases (COPD) or bronchial asthma (BA) in March. Their symptoms and HR-QoL were evaluated using a questionnaire including the SF-36v2 and COOP/WONCA charts in March, May and July. PM2.5 was sampled in March and July in Ulaanbaatar, and its composition was analyzed.

**Results:**

Patients with COPD or BA showed higher prevalence of respiratory symptoms than the control subjects in each month. For HR-QoL, all subscales worsened in the patients than in the control group in March. Although the HR-QoL of the COPD and control groups were not significantly changed through the surveys, some subscales of the BA group showed remarkable improvement in July as compared to March. Daily means of PM2.5 in March were significantly higher than those in July. Carbon and ionic component concentrations, except for magnesium and calcium ions, were significantly higher in March than July. Mass concentrations of some metallic components were also significantly higher in March than July. The percentage of nitrate ion in PM2.5 was significantly higher in March when compared to that in July.

**Conclusions:**

These results suggested that the symptoms in the COPD and BA groups were caused by the disease, and the association with air pollution or seasonality remained unclear. However, the effects of air pollution and seasonality on the HR-QoL were significant in the patients with BA.

**Electronic supplementary material:**

The online version of this article (doi:10.1186/s40064-016-3481-x) contains supplementary material, which is available to authorized users.

## Background

Ulaanbaatar, Mongolia is reported to be one of the most air-polluted cities in the world (WHO 2014). One of the reasons is attributable to the increased coal consumption during the winter months, as well as geographical characteristics that Ulaanbaatar is surrounded by mountains (basin) (Amarsaikhan et al. 2014). Recently, population influx from the rural area to Ulaanbaatar has increased, leading to development of the district dense with “ger”, which is a Mongolian traditional dwelling. The residents of ger district use coal as household fuels due to poor infrastructure. Dashdendev et al. (2011) reported that the children living in urban area showed lower pulmonary function when compared to the children living in the rural area, and they concluded that the deterioration of the pulmonary function reflected the air pollution. However, the reports on health effects of environment intended to adult, the elderly, or patients with chronic respiratory diseases (CRD) were insufficient. We previously performed a survey for the general population aged 40–79 years in Ulaanbaatar, and found that the respiratory symptoms and health-related quality of life (HR-QoL) in the subjects with ventilatory impairments living in the ger district worsened in the cold season. PM2.5 and PM10 levels were significantly higher in the cold season than in the warm season, and 90 % of the subjects living in the ger district used smoke-rich fuel for heating. These results suggested that high PM2.5 level exacerbates respiratory symptoms and the HR-QoL of subjects with ventilatory impairments in the cold season. Particulate matters have been reported to be risk factors of cardiopulmonary diseases among the susceptible groups such as children, elderly people, and patients with cardiopulmonary disorders (Pope 2000). Recently, Lim et al. (2012) reported that five principal risk factors, including ambient particulate matter and air pollution, are involved in Disabled-Adjusted Life Years (DALY), which is a measure of overall disease burden expressed as the number of years lost due to ill health, disability, or early death. These studies suggested that PM2.5 is involved in the onset and exacerbation of cardiopulmonary diseases by acute and chronic exposures. Enkhjargal et al. (2008) reported that outpatient visits increased when air pollution was severe in the ger district during winter months in Ulaanbaatar. However, there are few research studies targeting the Mongolian susceptible group, which consists of adult patients with CRD. Therefore, we conducted a survey targeted at patients with COPD and BA in Ulaanbaatar to examine the effects of PM2.5 air pollution and seasons on respiratory symptoms and HR-QoL.

## Methods

### Subjects

Subjects were 82 patients who visited the Department of Respiratory Medicine in the affiliated hospital of the Mongolian National University of Medical Sciences in March 2012 and 2013. Patients with COPD and BA aged 40–79 years and in stable state (not in exacerbation state) were classified as “Patients”, whereas hospital patients without any respiratory diseases but with a moderate disease such as mild hypertension were recruited as “Controls”. The patients and hospital controls filled out the questionnaire three times in March, May and July. Respiratory diseases were diagnosed by respiratory specialist and pulmonary function test. The pulmonary function test was performed using spirometer HI-105 (CHEST M.I., Tokyo, Japan) and the subjects were classified into four groups according to the guidelines of The Japanese Respiratory Society; normal, obstructive, restrictive, and combined ventilatory impairment (Sasaki et al. 2001). Patients were classified as COPD or BA according to the clinical findings, spirometry results, and the onset and duration of symptoms. Patients and controls with infection or severe comorbidities such as cancer or pneumonectomy were excluded. We also excluded subjects who did not fill out 3 consecutive questionnaires and those who did not take the pulmonary function test. In total, 27 patients (16 with COPD and 11 with BA) and 27 controls were included in this study.

### Questionnaire

The questionnaire is a self-completed booklet containing questions on the age, sex, smoking history, occupation, respiratory symptoms: Q1, Does the weather affect your cough?; Q2, Have you ever coughed up sputum from your chest when you do not have a cold?; Q3, Do you usually cough up sputum from your chest first thing in the morning?; Q4, How frequently do you wheeze? SF-36v2 and COOP/WONCA charts. Responses to SF-36 were on three-, five- or six-point ordinal scales, from which 8 subscales (physical functioning, PF; role limitations due to physical health problems, RP; bodily pain, BP; general health perceptions, GH; vitality, VT; social functioning, SF; role limitations due to emotional problems, RE; mental health, MH) were calculated from 0 to 100 points (min = 0, max = 100) according to the scoring manual (Fukuhara et al. 2004). Responses to the COOP/WONCA charts comprising of 8 items (physical fitness, PF; feelings, FE; daily activities, DA; social activities, SA; change of health, CH; overall health, OH; pain, PA; quality of life, QL) were scored on a five-point ordinal scale ranging from 1 to 5 (1 = best, 5 = worst) (van Weel 1993).

### Measurement of ambient PM2.5 level and the analyses of their components

Ambient air was sampled in the center of Ulaanbaatar city at 47°54′53.0″N 106°55′24.2″E, away from heavy traffic and any smoke-generating facility. Fine particles with a diameter of 2.5 μm or less (PM2.5) were collected on polytetrafluoroethylene (PTFE) filters using FRM-2000 (Thermo Fisher Scientific Inc., Waltham, MA, USA) at a flow-rate of 16.7 L/min. Sampling times were 9 am to 4:50 pm and 5 pm to 8:50 am of the next morning. Personal exposure level of PM2.5 was measured using mini pump MP-∑3 (SIBATA SCIENTIFIC TECHNOLOGY, Tokyo, Japan) equipped with PTFE filter at a flow-rate of 2.50 L/min. Sampling time for mini pump was 9 am to 5 pm. Sampled filters were stored in hermetically sealed plastic bags at −20 °C. Filters were weighed, followed by conditioning at 21.5 ± 1.5 °C and 35.0 ± 5.0 % relative humidity using an electric analytical scale (ME-5F, Sartorius AG, Göttingen, Germany). PM2.5 mass concentration was calculated by subtracting the filter weight of pre-sampling from post-sampling. Carbon and ionic components contained in the particles sampled on the filters were analyzed by FUJITSU QUALITY LABORATORY (Kawasaki, Japan).

### Data handling and statistical analyses

All data were handled by questionnaire IDs and managed as electronic data for the analysis. Statistical analyses were performed using the statistical software packages JMP version 11 (SAS Institute, Cary, NC, USA), including Welch’s *t* test and one-way analysis of variance (ANOVA) followed by Tukey’s HSD test for the parametric analyses of continuous variables of two and three groups respectively, and χ^2^-test for analyses of categorical data. *P* values <0.05 were considered significant.

### Ethical considerations

The present study was approved by the Clinical Ethical Review Board of Kurume University School of Medicine. Before investigation, all participants were given explanations in person as to the purpose and method of the study, as well as information regarding handling of the results. The study was carried out upon receipt of written consent.

## Results

### Characteristics of participated patients

Table 1 shows the demographic characteristics of the participants. There were no significant differences in the age between each group. In contrast, all patients with BA were females and the male-to-female ratio was statistically significant between each group. There were no current or ex-smokers in the BA group, and only one current and one ex-smoker in the control group. However, more than half (62.5 %) of the subjects in the COPD group were current or ex-smokers. Majority of the patients with COPD or BA were not in employment whereas approximately 60 % of the control subjects were employed.

**Table 1:** Demographic characteristics of participants

	Control	COPD	BA	Statistical analysis
n	27	16	11	Welch’s *t* test
Age (years, mean ± SD)	55.7 ± 10.4	58.9 ± 8.1	57.8 ± 11.9	N.S.
[Min–max]	40–76	48–76	41–78	
Sex [n (%)]				χ^2^-test
Male	8 (29.6)	9 (56.3)	0 (0.0)	*P* < 0.01
Female	19 (70.4)	7 (43.8)	11 (100.0)	
Smoking status [n (%)]				
Current smoker	1 (3.9)	6 (37.5)	0 (0.0)	*P* < 0.0005
Ex-smoker	1 (3.9)	4 (25.0)	0 (0.0)	
Never smoker	24 (92.3)	6 (37.5)	9 (81.8)	
Unknown	1 (3.9)	0 (0.0)	2 (18.2)	
Occupation [n (%)]				
White collar	8 (29.6)	1 (6.3)	0 (0.0)	*P* < 0.01
Blue collar	8 (29.6)	1 (6.3)	1 (9.1)	
Not in employment	11 (40.7)	14 (87.6)	10 (90.9)	

Data are presented as mean ± standard deviation (SD) and range between the minimum and maximum values (Min–Max). Categorical variables were presented as the sample number and its percentage (n (%)). Statistical analyses were performed using Welch’s *t* test for age and χ^2^-test for categorical variables. *P* value less than 0.05 was considered as statistically significant

*COPD* chronic obstructive pulmonary disease, *BA* bronchial asthma

### Respiratory symptoms

Subjects were queried about respiratory symptoms. The prevalence of these symptoms were significantly higher in all three surveys (March, May, and July) in the COPD or BA patients than those in the control group (Table 2), in which the prevalence was not significantly changed by month. The number of the symptoms was also higher in the COPD and BA groups than that in the control group in each survey. There were no significant differences between the surveys in each group.

**Table 2:** Prevalence of respiratory symptoms in each month

	March			*P* value	May			*P* value	July			*P* value
	Control	COPD	BA	χ^2^-test	Control	COPD	BA	χ^2^-test	Control	COPD	BA	χ^2^-test
Q1 [n, (%)]	3 (11.1)	8 (50.0)	6 (60.0)	<0.005	7 (25.9)	8 (50.0)	9 (90.0)	<0.005	7 (26.9)	11 (68.8)	9 (81.8)	<0.005
Q2 [n, (%)]	8 (26.3)	10 (62.5)	7 (63.6)	<0.05	7 (25.9)	10 (62.5)	7 (70.0)	<0.05	5 (19.2)	11 (68.8)	7 (63.4)	<0.05
Q3 [n, (%)]	3 (11.1)	10 (62.5)	4 (36.4)	<0.005	3 (11.1)	8 (50.0)	7 (70.0)	<0.001	3 (11.5)	7 (43.8)	5 (50.0)	<0.001
Q4 [n, (%)]	5 (18.5)	11 (68.8)	11 (100.0)	<0.0001	4 (14.8)	12 (75.0)	8 (80.0)	<0.0001	3 (12.0)	13 (81.3)	7 (63.6)	<0.0001
Number of symptoms	0.70 ± 0.99	2.44 ± 1.36 *P* < 0.0001 Versus control	2.55 ± 1.13 *P* < 0.0005 Versus control		0.78 ± 1.12	2.38 ± 1.41 *P* < 0.001 Versus control	2.82 ± 1.54 *P* < 0.0005 Versus control		0.67 ± 1.00	2.63 ± 0.89 *P* < 0.0001 Versus control	2.55 ± 1.21 *P* < 0.0001 versus Control	

Questions regarding respiratory symptoms: Q1, Does the weather affect your cough?; Q2, Do you ever cough up sputum from your chest when you don’t have a cold?; Q3, Do you usually cough up sputum from your chest first thing in the morning?; Q4, How frequently do you wheeze? Number of symptoms were presented as mean ± SD, and statistical analyses were performed using one-way analysis of variance (ANOVA) followed by Tukey’s HSD test. *P* value less than 0.05 was considered as statistically significant

### HR-QoL measured by the SF-36v2 and COOP/WONCA charts

**Fig. 1:**
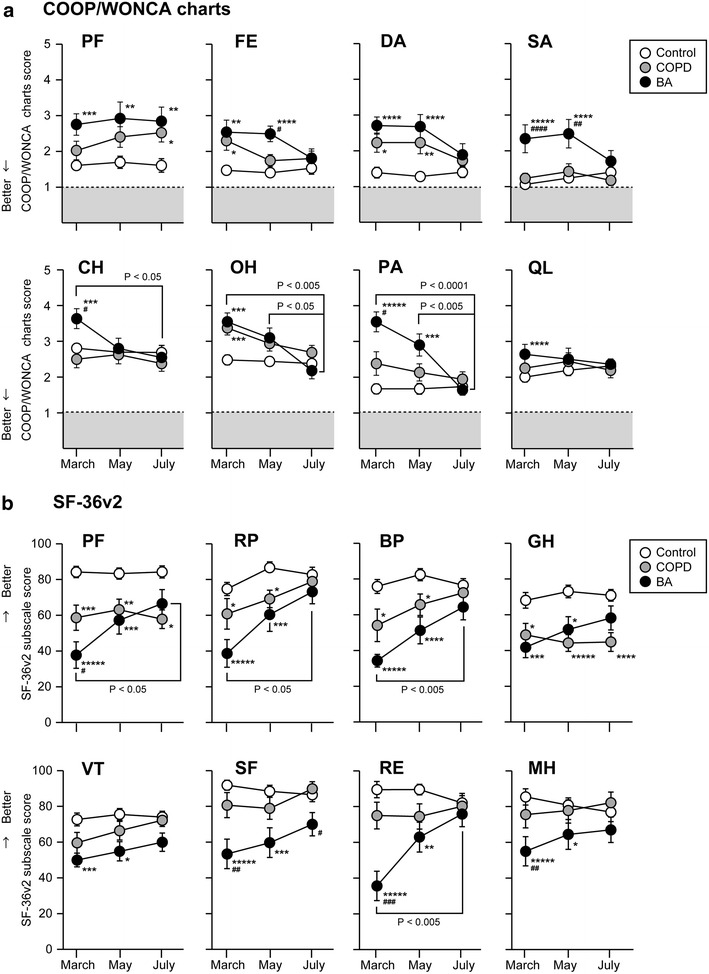
Seasonal changes of the subscale scores of the HR-QoL measured by the COOP/WONCA charts (**a**) and SF-36v2 (**b**) in the COPD, BA and control groups. The raw scores of SF-36v2 were converted to subscale scores. Data are presented as mean ± standard error of the mean (Additional file 1: Table S1). The COOP/WONCA charts items were represented as follows: physical fitness, PF; feelings, FE; daily activities, DA; social activities, SA; change in health, CH; overall health, OH; pain, PA; quality of life, QL. The subscales of SF-36v2 were represented as follows; physical functioning, PF; role limitations due to physical health problems, RP; bodily pain, BP; general health perceptions, GH; vitality, VT; social functioning, SF; role limitations due to emotional problems, RE; and mental health, MH. * *P* < 0.05; **P < 0.01; ***P < 0.005; ****P < 0.001; *****P < 0.0001 versus control. ^#^ *P* < 0.05; ^##^ *P* < 0.01; ^###^ *P* < 0.005; ^####^ *P* < 0.001 versus COPD group

In March, the BA group showed significantly worse scores of all subscales of the SF-36v2 and all items of the COOP/WONCA charts than those in the control subjects (Fig. 1, Additional file 1: Table S1). The COPD group showed significantly worse scores of the FE, DA and OH items of the COOP/WONCA charts and the PF, RP, BP and GH subscales of SF-36v2 than those in the control group in March. In May, the PF, FE, DA, SA and PA items of the COOP/WONCA charts and all subscales of SF-36v2 were significantly worse in the BA group than those in the control group. The DA item of the COOP/WONCA charts and the PF, RP, BP and GH subscales of SF-36 were significantly worse in the COPD group than those in the control group. In July, PF of the COOP/WONCA charts and SF of the SF-36v2 were significantly worse in the BA group than in the control group. The COPD group scored significantly worse in the PF of the COOP/WONCA charts and the PF and GH of SF-36v2 than those in the control group. In the BA group, the SA, CH and PA of the COOP/WONCA charts in March, as well as the FE and SA in May were significantly worse than those in the COPD group in the same month. For the subscale of SF-36v2, the BA group scored significantly worse scores of the PF, SF, RE and MH in March when compared to those in the COPD group. In the control and COPD groups, there were no significant differences in the COOP/WONCA charts and SF-36v2 subscale scores between each month. In contrast, the BA group showed significant improvements in July in the CH, OH and PA of the COOP/WONCA charts and the PF, RP, BP and RE of SF-36v2 when compared to those in March. Significant improvements were also observed between May and July in the scores of the OH and PA items of the COOP/WONCA charts in the BA group.

### Ambient air pollution in Ulaanbaatar, Mongolia

PM2.5 was measured in Ulaanbaatar (Table 3). Daily means of PM2.5 in March [60.3 ± 15.5 (mean ± SD, min–max: 40.7–81.5) μg/m^3^] were significantly higher than those in July [9.7 ± 2.6 (6.6–12.7) μg/m^3^]. Organic and elemental carbon, ionic, and metallic components were also measured. Carbon and ionic component concentrations, except for K^+^, Mg^2+^ and Ca^2+^, were significantly higher in March than July. Mass concentrations of metallic components such as V, Cr, Fe, Co, Ni, Cu, Zn, As, Se, Rb, Mo, Sb, Cs and W were also significantly higher in March than July.

**Table 3:** Difference of the components of PM2.5 between March and July sampled in Ulaanbaatar, Mongolia

	March			July			*P* value
	Mean ± SD	Median	Min–max	Mean ± SD	Median	Min–max	
*Mass concentration (μg/m* ^*3*^ *)*							
PM2.5	60.29 ± 15.48	58.71	40.74–81.49	9.72 ± 2.59	10.52	6.57–12.71	<0.0005
*Carbon (μgC/m* ^*3*^ *)*							
Organic	21.47 ± 7.00	18.78	13.51–31.93	2.22 ± 0.95	2.63	080–3.29	<0.005
Elemental	7.72 ± 1.68	7.39	5.80–10.77	1.52 ± 0.57	1.61	0.75–2.15	<0.0005
Total	29.19 ± 8.58	26.18	19.36–42.70	3.73 ± 1.50	4.22	1.54–5.19	<0.001
*Ion (μg/m* ^*3*^ *)*							
Cl^−^	0.710 ± 0.22	0.646	0.556–1.176	0.0302 ± 0.0197	0.0254	0.0106–0.0716	<0.001
NO_3_^−^	2.60 ± 0.86	2.77	1.49–3.47	0.0496 ± 0.0477	0.0471	N.D.–0.1169	<0.001
SO_4_^2−^	3.83 ± 0.98	4.37	2.26–4.58	0.5260.156	0.522	0.764	<0.0005
Na^+^	0.0846 ± 0.0194	0.0846	0.0604–0.1084	0.0183 ± 0.0100	0.0174	0.0085–0.0390	<0.0005
NH_4_^+^	2.80 ± 0.65	3.04	1.71–3.42	0.206 ± 0.072	0.194	0.115–0.317	<0.0005
K^+^	0.0242 ± 0.0250	0.0138	0.0024–0.0637	0.000681 ± 0.001370	N.D.	N.D.–0.003639	N.S.
Mg^2+^	N.D.	N.D.	N.D.	N.D.	N.D.	N.D.	–
Ca^2+^	0.290 ± 0.064	0.265	0.227–0.371	0.262 ± 0.098	0.284	0.111–0.407	N.S.
*Metal (ng/m* ^*3*^ *)*							
Na	111.75 ± 39.91	100.84	65.86–165.06	67.57 ± 109.06	28.03	16.03–314.64	N.S.
Al	113.10 ± 68.34	115.55	34.11–204.03	62.65 ± 90.09	21.68	N.D.–234.25	N.S.
Si	108.74 ± 47.95	111.34	46.51–163.46	89.81 ± 84.82	56.57	20.96–266.00	N.S.
K	116.17 ± 35.86	101.89	74.88–172.71	112.97 ± 154.26	51.86	41.74–461.23	N.S.
Ca	315.09 ± 149.22	265.58	193.68–559.36	192.02 ± 106.51	172.92	80.57–392.83	N.S.
Sc	0.0184 ± 0.0076	0.0203	0.0050–0.0257	0.0104 ± 0.0079	0.0079	0.0026–0.0245	N.S.
Ti	6.16 ± 2.60	4.95	3.69–10.14	4.93 ± 2.77	3.42	3.20–10.16	N.S.
V	0.482 ± 0.134	0.485	0.267–0.637	0.174 ± 0.110	0.137	0.061–0.402	<0.005
Cr	14.36 ± 10.81	12.777	N.D.–28.059	0.772 ± 1.787	N.D.	N.D.–4.793	<0.05
Mn	4.80 ± 1.05	4.96	3.48–6.20	4.28 ± 1.81	3.74	2.29–7.58	N.S.
Fe	217.82 ± 114.46	276.11	67.41–322.63	86.65 ± 43.46	87.67	34.73–167.96	<0.05
Co	0.115 ± 0.034	0.112	0.060–0.157	0.0455 ± 0.0265	0.0339	0.0285–0.1029	<0.005
Ni	0.469 ± 0.203	0.448	0.154–0.778	0.110 ± 0.049	0.096	0.056–0.184	<0.01
Cu	4.68 ± 2.68	3.82	2.51–9.69	1.73 ± 0.46	1.52	1.08–2.33	<0.05
Zn	53.02 ± 27.04	51.33	27.64–101.61	18.14 ± 17.19	12.25	2.82–49.45	<0.05
As	14.14 ± 4.77	13.97	8.39–22.61	0.409 ± 0.267	0.33	0.21–0.99	<0.001
Se	0.233 ± 0.092	0.234	0.106–0.349	0.0724 ± 0.0386	0.0562	0.0384–0.1441	<0.01
Rb	0.338 ± 0.068	0.330	0.265–0.425	0.162 ± 0.062	0.138	0.114–0.286	<0.001
Mo	0.133 ± 0.071	0.131	0.033–0.227	0.0352 ± 0.0319	0.0225	0.0133–0.1031	<0.05
Sb	0.988 ± 0.422	1.065	0.412–1.414	0.466 ± 0.277	0.621	0.114–0.800	<0.05
Cs	0.0816 ± 0.0276	0.0771	0.0511–0.1308	0.0197 ± 0.0108	0.0145	0.0120–0.0388	<0.005
Ba	29.84 ± 39.18	6.74	3.44–95.01	3.31 ± 2.74	2.15	1.82–9.43	N.S.
La	0.0955 ± 0.0383	0.0795	0.0626–0.1445	0.198 ± 0.171	0.150	0.050–0.512	N.S.
Ce	0.197 ± 0.077	0.169	0.128–0.291	0.143 ± 0.076	0.109	0.072–0.288	N.S.
Sm	0.0163 ± 0.0077	0.0127	0.0099–0.0261	0.0110 ± 0.0073	0.0077	0.0059–0.0255	N.S.
Hf	0.00293 ± 0.00170	0.00266	0.00090–0.00588	0.00151 ± 0.00079	0.00116	0.00066–0.00301	N.S.
Ta	0.000345 ± 0.000387	0.000290	N.D.–0.001039	0.000040 ± 0.000105	N.D.	N.D.–0.000278	N.S.
W	0.211 ± 0.169	0.141	0.034–0.462	0.0220 ± 0.0299	0.0076	0.0070–0.0883	<0.05
Pb	12.34 ± 5.73	10.41	8.29–23.56	12.27 ± 11.42	6.92	2.67–34.15	N.S.
Th	0.0232 ± 0.0152	0.0174	0.0083–0.0459	0.00964 ± 0.00633	0.00675	0.00522–0.02305	N.S.

Data are presented as mean ± standard deviation (SD), median, and range between the minimum and maximum values (Min–Max). Statistical analyses were performed using Mann–Whitney’s *u* test. *P* value less than 0.05 was considered as statistically significant. N.D., not detected; N.S., not significant

## Discussion

We investigated the effects of air pollution and seasons on the respiratory symptoms and the HR-QoL of Mongolian patients with CRD, who were susceptible to air pollution. The symptoms were more prevalent, and the HR-QoL was worse in the CRD patients when compared to those in the controls. The symptoms and the HR-QoL of the CRD patients improved in concordance with warmer temperatures and reduced air pollution. In some subscales of the HR-QoL, BA patients showed significantly worse scores than those of the COPD patients. Some subscales of the HR-QoL of BA patients showed significant improvements over the surveys. Effect of NO_3_^−^ present in PM2.5 seemed to affect HR-QoL because the percentage of NO_3_^−^ was significantly higher in March (4.5 % of PM2.5) when compared to that in July (0.4 %). There were few reports targeting adult patients aged >40 years with CRD in Ulaanbaatar, Mongolia. Therefore, this is the first study on the health effects of air pollution and seasons on Mongolian adult patients with COPD or BA.

There are many published reports regarding the effects of PM2.5 on the HR-QoL of adults, especially for patients with CRD (Ni et al. 2015; Atkinson et al. 2014; Weichenthal et al. 2013). The WHO guideline for 24-h average of PM2.5 is 25 μg/m^3^ (WHO 2005). All-cause mortality was reported to increase at 3 % per 25 μg/m^3^ increase of PM2.5, and Burnett and Goldberg similarly reported that all-cause mortality increased 2.2 % per 25 μg/m^3^ increase of PM2.5 (Klemm and Mason 2003; Burnett and Goldberg 2003). Pope reported that hospitalization from COPD and BA increased approximately 2.5 and 2 % respectively, while prevalence of the lower respiratory and asthmatic symptoms increased approximately 3 % per 5 μg/m^3^ increase of PM2.5 acute exposure. As for the chronic effect, 2.5 μg/m^3^ increase of PM2.5 caused the elevation of cardiopulmonary mortality and the prevalence of bronchitis at 5.5 and 7 % respectively (Pope 2000). Emergency visit due to asthma was reported to be correlated with air pollution (Sun et al. 2006). Abe et al. reported that increased NOx and CO concentrations correlated with emergency department visit due to adult asthma, along with the temperature and humidity of the environment, although multiple regression analysis showed no significant correlation in the temperature in Japan (Abe et al. 2009). On the other hand, Rossi et al. (1993) reported that the temperature was significantly associated with hospital due to asthma attack. A seasonality in emergency visit due to asthma was reported in some preceding studies but results are inconsistent (Bates et al. 1990; Goldstein and Currie 1984). Collectively, these reports suggested that exacerbation of asthma differs according to geographic areas because the climate, weather, and other atmospheric conditions were varied. There remains one question on whether air pollution has independent effects on respiratory health. Rossi et al. (1993) showed the correlation between NO_2_, H_2_S and asthma visits after standardization of temperature, which suggested that air pollution was an independent risk factor for the exacerbation of asthma. Tseng et al. (2013) demonstrated the effect of cold temperatures on the exacerbation of COPD, and Alahmari et al. (2015) reported that high level of atmospheric pollution had independent effects on the physical activity of COPD patients. Air pollution also increased the incidence of outpatient visits due to COPD even after standardization of temperature (To et al. 2015). These reports also suggested that air pollution had independent health impact on COPD and BA patients besides temperature.

Although the health effects of air pollution including mortality, morbidity, symptoms and physiological functions have been previously reported in Mongolia (Nakao et al. 2016), the concept of HR-QoL, which refers to the individual’s perception of well-being, should also be considered as an adverse health outcome (American Thoracic Society 2000). The effects of air pollution on the HR-QoL in adults with CRD were previously reported (Miravitlles et al. 2014; Jones et al. 2016). We therefore assessed the effect of season and CRD on HR-QoL in Mongolian subjects (Fig. 1, Additional file 1: Table S1). In Mongolia, combustion of fossil fuels seems to contribute to the increase of PM2.5, which affects the HR-QoL of Mongolian patients with COPD or BA. Organic carbon, Cl^−^, NO_3_^−^, NH_4_^+^ and K^+^ of PM2.5 showed significantly higher percentages in March than July. In particular, NO_3_^−^ was 10 times more in March than July. However, SO_4_^2−^ did not change significantly between March and July. These results suggested that NO_3_^−^ was mainly derived from the combustion of coal and further contributed to the deterioration of the HR-QoL of BA patients. Additionally, all subscales of the HR-QoL were worse in the CRD patients than in the control subjects in March. Although the HR-QoL of the COPD and control groups were not significantly changed over the surveys, some domains (the CH, OH, PA of the COOP/WONCA charts and the PF, RP, BP, GH, RE subscales of the SF-36v2) of the HR-QoL of the BA group showed remarkable improvements in July. The worsened HR-QoL during winter was ameliorated by the reduction of air pollution in association with an increase in temperature due to seasonal change. To reduce air pollution in Ulaanbaatar, it is necessary to replace heating and cooking equipment to smoke-free equipment. With respect to the respiratory symptoms, the prevalence in March was higher in the CRD patients than in the control subjects and remained high in July (Table 2). These results suggested that the symptoms in the patients with CRD were caused by the disease itself and the worse score on daily living activity was affected by air pollution independently from the symptoms. The associations between respiratory symptoms and PM2.5 air pollution or seasonality in Mongolia have not yet been clarified.

Outdoor personal PM2.5 exposure levels were [104.7 ± 27.9 (mean ± SD, min–max: 58.3–166.3) μg/m^3^] in March and [19.9 ± 15.1 (5.3–61.3) μg/m^3^] in July, while indoor personal exposure levels were [49.5 ± 16.8 (13.4–69.2) μg/m^3^] in March and [11.8 ± 6.4 (2.5–18.6) μg/m^3^] in July (data not shown). These results indicated that the indoor level of PM2.5 was higher when the outdoor PM2.5 was high, even though the room was not ventilated. It is recommended to avoid unnecessary outings for susceptible people, such as patients with COPD or BA. As for public administration, it is recommended to set forward a widespread use of high-efficient heating systems and introduction of environmentally friendly power plants.

There were a few limitations in this study. Firstly, the sample number was small because this study was a pilot study to examine the symptoms and HR-QoL of patients with CRD. Secondly, we used easy-to-understand yes–no questions regarding the respiratory symptoms in priority to a more detailed assessment. This could be the reason why differences between each month were not found in any questions for the symptoms. Finally, the health impact of air pollution and temperature was difficult to distinguish and to estimate each risk for the conditions of the patients. It was also necessary to perform multivariate analyses, but was not possible due to the small sample number and limitation of the information. Therefore, it is crucial to collect detailed basic and medical information from larger sample sizes in further studies.

## Conclusions

We investigated the effects of air pollution and seasons on the symptoms and HR-QoL in patients with CRD in Ulaanbaatar. The patients showed higher prevalence of respiratory symptoms than those in the control group. All subscales of the HR-QoL were worse in the patients than in the control group in March. Although the HR-QoL of the COPD and control groups were not significantly changed over the surveys, some subscales of the HR-QoL of the BA group showed remarkable improvements in July. PM2.5, which was reported to cause exacerbation of COPD and BA, was significantly severe in March than July. These results suggested that the symptoms in the patients were caused by the disease itself and their association with PM2.5 or seasonality have not yet been clarified. However, the effects of PM2.5 and seasons on the HR-QoL were significant in patients with BA.

## Additional file

**Additional file 1.** Seasonal changes of the subscale scores of the HR-QoL measured by the COOP/WONCA charts and SF-36v2 in the COPD, BA and control groups.

## Abbreviations

HR-QoL: health-related quality of life
COPD: chronic obstructive pulmonary disease
BA: bronchial asthma
CRD: chronic respiratory diseases
DALY: disabled-adjusted life years
SF-36: short-form 36 health survey
PF: physical functioning (subscale of the SF-36) or physical fitness (item of the COOP/WONCA charts)
RP: role limitations due to physical problems
BP: bodily pain
GH: general health perceptions
VT: vitality
SF: social functioning
RE: role limitations due to emotional problems
MH: mental health
FE: feelings
DA: daily activities
SA: social activities
CH: change of health
OH: overall health
PA: pain
QL: quality of life
PTFE: polytetrafluoroethylene
ANOVA: analysis of variance
