# Supplementary material for: Effects of air pollution and seasonality on the respiratory symptoms and health-related quality of life (HR-QoL) of outpatients with chronic respiratory disease in Ulaanbaatar: pilot study for the comparison of the cold and warm seasons
Source: Springerplus. 2016 Oct 19;5(1):1817. doi: 10.1186/s40064-016-3481-x (PMC5069213; doi:10.1186/s40064-016-3481-x)
Supplement: Supplementary file 1 — Additional file 1. Seasonal changes of the subscale scores of the HR-QoL measured by the COOP/WONCA charts and SF-36v2 in the COPD, BA and control groups. [file 40064_2016_3481_MOESM1_ESM.docx]

|  | March | | |  | May | | |  | July | | |
| --- | --- | --- | --- | --- | --- | --- | --- | --- | --- | --- | --- |
| COOP/WONCA  charts | Control  [n = 27] | COPD  [n = 16] | BA  [n = 11] |  | Control  [n = 27] | COPD  [n = 16] | BA  [n = 11] |  | Control  [n = 27] | COPD  [n = 16] | BA  [n = 11] |
| PF | 1.6 ± 0.6 | 2.0 ± 1.0 | 2.7 ± 1.0^***^ |  | 1.7 ± 0.9 | 2.4 ± 1.2 | 2.9 ± 1.5^**^ |  | 1.6 ± 1.0 | 2.5 ± 1.0^*^ | 2.8 ± 1.3^**^ |
| FE | 1.5 ± 0.7 | 2.3 ± 1.1^*^ | 2.6 ± 1.1^**^ |  | 1.4 ± 0.6 | 1.8 ± 0.7 | 2.5 ± 0.7^****, #^ |  | 1.5 ± 0.9 | 1.8 ± 0.8 | 1.8 ± 0.9 |
| DA | 1.4 ± 0.8 | 2.3 ± 1.1^*^ | 2.7 ± 0.8^****^ |  | 1.3 ± 0.6 | 2.3 ± 1.2^**^ | 2.7 ± 1.1^****^ |  | 1.4 ± 0.8 | 1.8 ± 1.1 | 1.9 ± 1.0 |
| SA | 1.1 ± 0.3 | 1.3 ± 0.6 | 2.4 ± 1.3^*****, #####^ |  | 1.3 ± 0.6 | 1.4 ± 0.9 | 2.5 ± 1.3^****, ##^ |  | 1.4 ± 0.7 | 1.2 ± 0.5 | 1.7 ± 1.0 |
| CH | 2.8 ± 0.7 | 2.5 ± 1.0 | 3.4 ± 0.9^***, #, ¶^ |  | 2.7 ± 0.9 | 2.6 ± 1.0 | 2.8 ± 0.9 |  | 2.7 ± 1.0 | 2.4 ± 0.9 | 2.6 ± 0.8 |
| OH | 2.5 ± 0.8 | 3.4 ± 0.8^***^ | 3.6 ± 0.8^***, ¶¶¶^ |  | 2.4 ± 0.7 | 2.9 ± 0.9 | 3.1 ± 0.9^¶^ |  | 2.4 ± 0.9 | 2.7 ± 0.8 | 2.2 ± 0.8 |
| PA | 1.7 ± 0.7 | 2.4 ± 1.3 | 3.5 ± 0.9^*****, #, ¶¶¶¶¶^ |  | 1.7 ± 0.8 | 2.1 ± 1.0 | 2.9 ± 1.0^***, ¶¶¶^ |  | 1.7 ± 0.7 | 1.9 ± 0.9 | 1.6 ± 0.5 |
| QL | 2.0 ± 0.5 | 2.3 ± 0.6 | 2.6 ± 0.7^****^ |  | 2.2 ± 0.6 | 2.4 ± 0.5 | 2.5 ± 0.9 |  | 2.3 ± 0.7 | 2.2 ± 0.4 | 2.4 ± 0.5 |
|  |  |  |  |  |  |  |  |  |  |  |  |
| SF-36v2 |  |  |  |  |  |  |  |  |  |  |  |
| PF | 84.2 ± 16.6 | 58.6 ± 23.4^***^ | 37.7 ± 24.6^*****, #, ¶^ |  | 83.3 ± 16.4 | 63.1 ± 23.4^**^ | 57.2 ± 23.2^***^ |  | 84.2 ± 17.3 | 67.8 ± 21.0^*^ | 66.5 ± 25.0 |
| RP | 84.7 ± 19.0 | 60.8 ± 28.4^*^ | 38.6 ± 25.7^*****, ¶^ |  | 86.6 ± 17.1 | 69.2 ± 19.6^*^ | 60.4 ± 28.1^***^ |  | 82.7 ± 21.4 | 78.9 ± 20.3 | 73.1 ± 21.1 |
| BP | 75.9 ± 20.2 | 54.1 ± 30.1^*^ | 34.3 ± 11.8^*****, ¶¶¶^ |  | 82.4 ± 17.5 | 65.6 ± 24.0^*^ | 51.2 ± 22.5^****^ |  | 76.5 ± 19.1 | 72.3 ± 23.0 | 64.3 ± 22.6 |
| GH | 68.0 ± 22.3 | 48.7 ± 21.4^*^ | 41.8 ± 19.3^***^ |  | 73.0 ± 18.1 | 44.2 ± 18.9^*****^ | 51.8 ± 21.6^*^ |  | 70.9 ± 16.7 | 44.8 ± 21.0^****^ | 58.2 ± 21.2 |
| VT | 72.7 ± 18.6 | 59.7 ± 19.2 | 50.0 ± 12.8^***^ |  | 75.5 ± 16.7 | 66.4 ± 20.9 | 54.9 ± 16.2^*^ |  | 74.0 ± 16.6 | 72.3 ± 13.9 | 60.0 ± 16.2 |
| SF | 91.9 ± 13.4 | 80.7 ± 23.3 | 53.4 ± 27.4^*****, ##^ |  | 88.4 ± 17.3 | 78.9 ± 24.5 | 59.7 ± 24.8^***^ |  | 86.5 ± 20.0 | 89.8 ± 16.0 | 70.0 ± 20.6^#^ |
| RE | 89.5 ± 21.8 | 75.0 ± 24.7 | 35.6 ± 27.2^*****, ###, ¶¶¶^ |  | 89.5 ± 15.4 | 74.5 ± 28.3 | 63.0 ± 25.0^**^ |  | 82.1 ± 27.2 | 80.2 ± 23.7 | 75.8 ± 22.4 |
| MH | 85.4 ± 12.7 | 75.6 ± 10.4 | 55.0 ± 18.4^*****, ##^ |  | 80.8 ± 15.7 | 77.8 ± 13.8 | 64.4 ± 19.1^*,^ |  | 76.9 ± 20.5 | 82.2 ± 15.5 | 67.0 ± 15.5 |

Supplementary table 1. Seasonal changes of the subscale scores of the HR-QoL measured by the COOP/WONCA charts and SF-36v2 in the COPD, BA and control groups

Data are presented as mean ± standard error of the mean. The COOP/WONCA charts items were represented as follows: physical fitness, PF; feelings, FE; daily activities, DA; social activities, SA; change in health, CH; overall health, OH; pain, PA; quality of life, QL. The subscales of SF-36v2 were represented as follows; physical functioning, PF; role limitations due to physical health problems, RP; bodily pain, BP; general health perceptions, GH; vitality, VT; social functioning, SF; role limitations due to emotional problems, RE; and mental health, MH. ^*^: P < 0.05; ^**^: P < 0.01; ^***^: P < 0.005; ^****^: P < 0.001; ^*****^: P < 0.0001 vs. control. ^#^: P < 0.05; ^##^: P < 0.01; ^###^: P < 0.005; ^####^: P < 0.001 vs. COPD group. ^¶^: P < 0.05; ^¶¶^: P < 0.01; ^¶¶¶^: P < 0.005; ^¶¶¶¶^: P < 0.001; ^¶¶¶¶¶^: P < 0.0001.
